# Molecular and phylogenetic characterization of honey bee viruses, *Nosema* microsporidia, protozoan parasites, and parasitic mites in China

**DOI:** 10.1002/ece3.464

**Published:** 2013-01-04

**Authors:** Bu Yang, Guangda Peng, Tianbang Li, Tatsuhiko Kadowaki

**Affiliations:** Department of Biological Sciences, Xi'an Jiaotong-Liverpool University111 Ren'ai Road, Suzhou Dushu Lake Higher Education Town, Jiangsu Province, 215123, China

**Keywords:** Chinese apiculture, honey bee pathogens and parasites, native Asian honey bees, nonnative European honey bees

## Abstract

China has the largest number of managed honey bee colonies, which produce the highest quantity of honey and royal jelly in the world; however, the presence of honey bee pathogens and parasites has never been rigorously identified in Chinese apiaries. We thus conducted a molecular survey of honey bee RNA viruses, *Nosema* microsporidia, protozoan parasites, and tracheal mites associated with nonnative *Apis mellifera ligustica* and native *Apis cerana cerana* colonies in China. We found the presence of black queen cell virus (BQCV), chronic bee paralysis virus (CBPV), deformed wing virus (DWV), Israeli acute paralysis virus (IAPV), and sacbrood virus (SBV), but not that of acute bee paralysis virus (ABPV) or Kashmir bee virus (KBV). DWV was the most prevalent in the tested samples. Phylogenies of Chinese viral isolates demonstrated that genetically heterogeneous populations of BQCV, CBPV, DWV, and *A. cerana*-infecting SBV, and relatively homogenous populations of IAPV and *A. meliifera*-infecting new strain of SBV with single origins, are spread in Chinese apiaries. Similar to previous observations in many countries, *Nosema ceranae,* but not *Nosema apis,* was prevalent in the tested samples. *Crithidia mellificae*, but not *Apicystis bombi* was found in five samples, including one *A. c. cerana* colony, demonstrating that *C. mellificae* is capable of infecting multiple honey bee species. Based on kinetoplast-encoded *cytochrome b* sequences, the *C. mellificae* isolate from *A. c. cerana* represents a novel haplotype with 19 nucleotide differences from the Chinese and Japanese isolates from *A. m. ligustica*. This suggests that *A. c. cerana* is the native host for this specific haplotype. The tracheal mite, *Acarapis woodi*, was detected in one *A. m. ligustica* colony. Our results demonstrate that honey bee RNA viruses, *N. ceranae*, *C. mellificae,* and tracheal mites are present in Chinese apiaries, and some might be originated from native Asian honey bees.

## Introduction

China has a long history (3000 years) of apiculture, and the native Asian honey bee, *Apis cerana cerana*, was the original major species used for apiculture, similar to that of other Asian countries (Chen 1993). From the early 20th century, colonies of the nonnative European honey bee, *Apis mellifera ligustica*, have been imported from different countries and distributed throughout China (Chen 1993); therefore, *A. c. cerana* and *A. m. ligustica* became the two major honey bee species maintained in Chinese apiaries. *A. c. cerana* colonies represented 90% of the total managed honey bee colonies in 1949; however, this reduced to 26.6% in 1991 (Li 1991; Food and Agriculture Organization of the United Nations (FAO) 2009). The number of managed *A. c. cerana* colonies has continued to decline because *A. c. cerana* is not suitable for migratory bee keeping and *A. c. cerana* is not able to produce royal jelly, which is the most valuable bee product in the Chinese apiculture industry. Thus, *A. m. ligustica* has become the dominant species maintained in Chinese apiaries.

The number of managed *A. mellifera* colonies has significantly declined in many countries in Europe and North America (Vanengelsdorp and Meixner 2010). In contrast, the number of managed *A. mellifera* colonies has increased over past decades in China. The managed honey bees in China grew from 3 million colonies in 1960 to 8.77 million colonies in 2008 (Xie 2011). Nevertheless, the colony losses have been observed in several regions (e.g., Shixing in Guangdong province) (Bu 2008). Although many factors are suggested for the loss of honey bee colonies throughout the world, pathogens and parasites are considered to have the major impacts on honey bee health (Vanengelsdorp and Meixner 2010; Williams et al. 2010).

Honey bees are exposed to various pathogens and parasites, such as viruses, bacteria, fungi, protozoans, and mites (Evans and Schwarz 2011). Viruses that have been identified to infect honey bees include acute bee paralysis virus (ABPV), black queen cell virus (BQCV), chronic bee paralysis virus (CBPV), deformed wing virus (DWV), Israeli acute paralysis virus (IAPV), Kashmir bee virus (KBV), and sacbrood virus (SBV), all of which are positive-strand RNA viruses (Chen and Siede 2007) belonging to the order Picornavirales (Le Gall et al. 2008). DWV, together with the infestation of the parasitic mite *Varroa destructor*, has been suggested to play a key role in winter colony losses (Dainat et al. 2012; Nazzi et al. 2012). *Paenibacillus larvae* and *Melissococcus plutonius* are the two major infective bacteria causing American and European foulbrood diseases, respectively (Forsgren 2010; Genersch 2010). The fungal pathogen *Ascosphaera apis* causes chalkbrood disease in larvae, thus weakening colony growth (Aronstein and Murray 2010). Two microsporidia, *Nosema apis* and *Nosema ceranae*, infect honey bees and may increase the nutritional requirement, morbidity, and mortality of the bee host (Martín-Hernández et al. 2011). Two protozoan parasites, *Crithidia mellificae* and *Apicystis bombi,* were also shown to infect honey bees (Langridge and McGhee 1967; Lipa and Triggiani 1996; Schmid-Hempel and Tognazzo 2010; Plischuk et al. 2011; Morimoto et al. 2012b). Although the roles of *C. mellificae* in honey bee health remain still unclear, a related species, *Crithidia bombi*, is one of the major parasites in bumble bees (Brown et al. 2003). Another protozoan parasite, the gregarine *A. bombi*, induces physical and behavioral effects on bumble bees, thereby shortening their life spans (Schmid-Hempel 2001; Rutrecht and Brown 2008). Nevertheless, its virulence in the honey bees is not well understood. Honey bee parasitic mites include *V. destructor*, *Acarapis woodi*, and *Tropilaelaps* spp., of which the former two are distributed throughout the world with the exception of a few countries, and the latter appears to be restricted to Asia (Anderson and Morgan 2007). *V. destructor* imposes dramatic negative impacts on honey bee health; therefore, it is considered a major threat to the apiculture industry in many countries (Rosenkranz et al. 2010).

China has the largest managed honey bee colonies in the world and the honey bee viruses were surveyed (Ai et al. 2012; Zhang et al. 2012); nevertheless, the epidemiological data on honey bee pathogens and parasites are still limited. We thus conducted a molecular survey of honey bee RNA viruses, *Nosema* microsporidia, protozoan parasites, and tracheal mites with both native *A. c. cerana* and nonnative *A. m. ligustica* colonies in China. The prevalence of each pathogen and parasite among the tested samples and the genetic diversity based on phylogenetic characteristics are discussed below.

## Materials and Methods

### Sample collection

*A. m. ligustica* colonies were sampled from 17 apiaries in seven provinces of mainland China in collaboration with the Apicultural Science Association of China (ASAC) from November 2011 to September 2012. Among the 17 apiaries, 11 were located at Suzhou, Wuxi, Yancheng, and Nanjing in Jiangsu province. Fifty worker bees were collected and pooled from a single colony from one apiary (17 colonies). Three *A. c. cerana* samples (each sample consisted of 50 pooled workers from a single colony) were collected from three managed colonies at three apiaries (SZ-3, SZ-5, and GD-2). Both *A. m. ligustica* and *A. c. cerana* colonies were simultaneously sampled at SZ-3 and SZ-5 apiaries. All of them appeared to be healthy colonies and the workers were collected from brood nests inside the hives. In addition, approximately 50 dying workers (crawling on the ground in front of the hive entrance) were sampled from *A. m. ligustica* colonies at eight apiaries.

*Varroa* and *Tropilaelaps* mites were collected at four apiaries, SZ-1, SZ-5, FJ (see Table 2), and an apiary in Wuxi when we detected the mites in colonies for sampling honey bees. They were collected from the same hives in which we sampled honey bees at SZ-1, SZ-5, and FJ. Thus, the infestation levels of mites at above apiaries were not determined. The mite species were identified by sequencing the parts of *cytochrome oxidase-1* genes, which were PCR-amplified with the following primer sets: 5′-CTATCCTCAATTATTGAAATAGGAAC-3′ and 5′-TAGCGGCTGTGAAATAGGCTCG-3′ for *Tropilaelaps mercedesae* (Anderson and Morgan 2007), and 5′-ATTTATTTTGATTTTTTGGRCA-3′ and 5′-AGCYCCTATWCTTAATACATA-3′ for *V. destructor* (Anderson and Fuchs 1998).

### RT-PCR detection of honey bee viruses

Total RNA was isolated from 20 workers collected from single colonies using Trizol reagent (Invitrogen). Total RNA (1 μg) was used for reverse transcription reaction using ReverTra Ace reverse transcriptase (TOYOBO) and a random primer. The reverse transcription products were then used for PCR with KOD FX DNA polymerase (TOYOBO) and the primer sets for detecting ABPV, BQCV, CBPV, DWV, IAPV, KBV, and SBV (Table 1). Honey bee *EF-1alpha* was used as a positive control to verify the quality of RNA extraction as well as reverse transcription, and also serves as an internal loading standard. The primer set, 5′-TGCAAGAGGCTGTTCCTGGTGA-3′ and 5′-CGAAACGCCCCAAAGGCGGA-3′, was used (Kojima et al. 2011a). *Varroa* and *Tropilaelaps* mite *Beta-actin* was also used as a positive control for RT-PCR of total RNA isolated from the mites. The primer set, 5′-TCGTACGAGCTTCCCGACGGT-3′ and 5′-GGGAGGCAAGGATGGAACCGC-3′, was used. These primer sequences were designed based on *V. destructor Beta-actin* gene sequence; however, they were successfully used to amplify *T. mersedesae Beta-actin* as well. The thermal cycling conditions were as follows: one cycle of initial denaturation at 94°C for 2 min, 35 cycles of denaturation at 98°C for 10 s, annealing at 55°C for 30 s, and extension at 68°C for 30 s. A negative control lacking template DNA and a positive DNA control were performed for each PCR reaction. Positive identification was confirmed by sequencing the PCR products. The PCR product was analyzed using 2% agarose gel electrophoresis.

**Table 1:** List of primers used for the detection and phylogenetic characterization of honey bee viruses in this study

Virus	Primer sequence	Length of amplicon	Reference
ABPV	5′-AATGGGCCTATGGACTTTTCTA-3′	178	Siede et al. (2008)
	5′-AAATCTCCTGCAATAACCTTGG-3′		
BQCV	5′-TGGTCAGCTCCCACTACCTTAAAC-3′	701	Benjeddou et al. (2001)
	5′-GCAACAAGAAGAAACGTAAACCAC-3′		
	5′-GTGGCGGAGATGTATGCGCTTTATC-3′	511	This study
	5′-CTGACTCTACACACGGTTCGATTAG-3′		
CBPV	5′-GACCCCCGTTGGAACGACGC-3′	233	Morimoto et al. (2012a)
	5′-CGGACGACGATTGGCGCTCA-3′		
	5′-TAYGAGYGATTTYTTGRGATCGAYTTCGCT-3′	335	This study
	5′-TGTAYTCGRCCTGATTRACGACRTTAGC-3′		
DWV	5′-ATTGTGCCAGATTGGACTAC-3′	435	Berényi et al. (2007)
	5′-AGATGCAATGGAGGATACAG-3′		
	5′-GCGAGCCAAATCAGGGCAAAACCTG-3′	820	This study
	5′-GGCGCGACCAAATCCACTCGACTGT-3′		
IAPV	5′-AAACATCACAGATGCTCAGGGTCGAGACTATATGT-3′	427	This study
	5′-CTAGGGAGCTACGGAGCGTGATTCGCCTTGTAGCT-3′		
	5′-GAAGGTTTGGGARGCYCCAYTWTGTAT-3′	706	This study
	5′-TGTTTGCRTCGGCHGTWGTTCCWGCAA-3′		
KBV	5′-ATGACGATGATGAGTTCAAG-3′	290	Shen et al. (2005)
	5′-AATTGCAAGACCTGCATC-3′		
SBV	5′-ACCAACCGATTCCTCAGTAG-3′	488	Grabensteiner et al. (2001)
	5′-CCTTGGAACTCTGCTGTGTA-3′		
	5′-AATGGTGCGGTGGACWATGGRGCAYGT-3′	558	This study
	5′-TGATACAGRGCRGCTCGRCARTTYTC-3′		

The bottom sets of primers were used for the phylogenetic characterization of BQCV, CBPV, DWV, IAPV, and SBV, respectively.

### Molecular phylogenetic analysis of honey bee viruses

To infer the phylogenies of Chinese BQCV, DWV, CBPV, IAPV, and SBV isolates, the following sequences were first determined: 511 nt sequences encoding a capsid protein of BQCV, 820 nt sequences encoding a capsid protein of DWV, 335 nt sequences encoding a putative RNA-dependent RNA polymerase (RdRP) of CBPV, 706 nt sequences encoding a part of RdRP and capsid protein of IAPV, and 558 nt sequences encoding a RdRP of SBV. The PCR products corresponding above sequences were obtained with the primer sets in Table 1. The parts of the viral genome sequences described above were chosen for analysis based on the availability of most sequences of other countries' viral isolates. After retrieving appropriate sequences from GenBank, all sequences were aligned using MUSCLE (Edgar 2004), and then the best-fit substitution model was selected for constructing each viral phylogeny. The selected model is described in the legends of Figs. 6. The phylogenetic tree was constructed using the maximum likelihood method and a bootstrap value of 1000 replicates with MEGA5 (Tamura et al. 2011) in each case.

**Figure 1:**
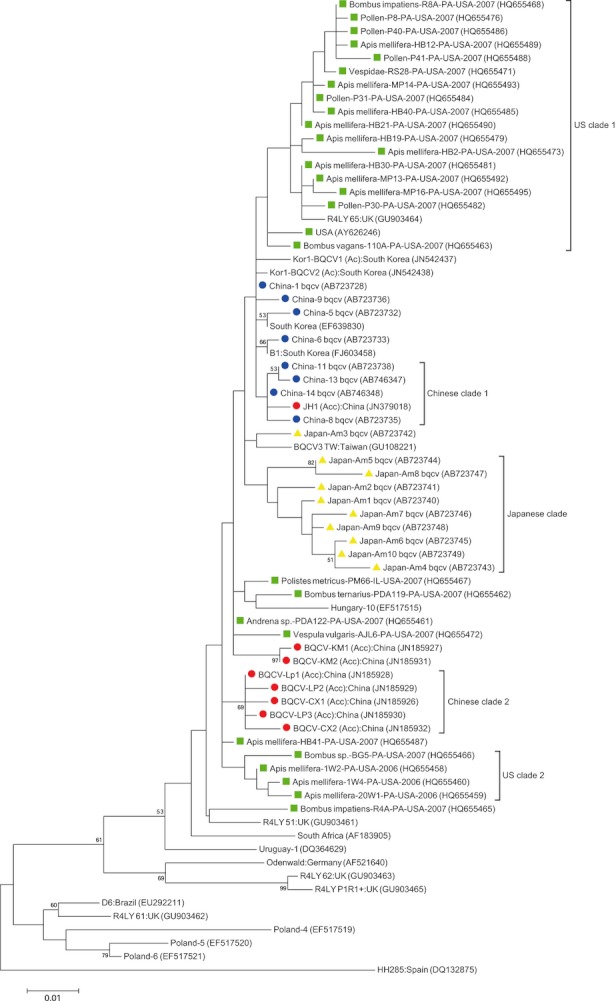
Phylogeny of black queen cell virus (BQCV) isolates from China and other countries. The phylogenetic tree based on alignment of the capsid protein gene sequences of BQCV isolates from various countries was constructed using the maximum likelihood method under the Tamura 3-parameter with a discrete gamma distribution and invariable sites model (T92 + G+I). The indicated branching topology was evaluated by bootstrap resampling of the sequences 1000 times, and nodes supported by bootstrap values >50 are shown. Each isolate is indicated by country of isolation and GenBank accession number. Chinese isolates identified by this study are shown by blue circles. Chinese isolates identified by other studies are indicated by red circles, and were isolated from *Apis cerana cerana* (Acc). The US and Japanese isolates are indicated by green squares and yellow triangles, respectively. The Japanese clade, Chinese clade 1 and clade 2, and US clade 1 and clade 2 are shown by brackets.

**Figure 2:**
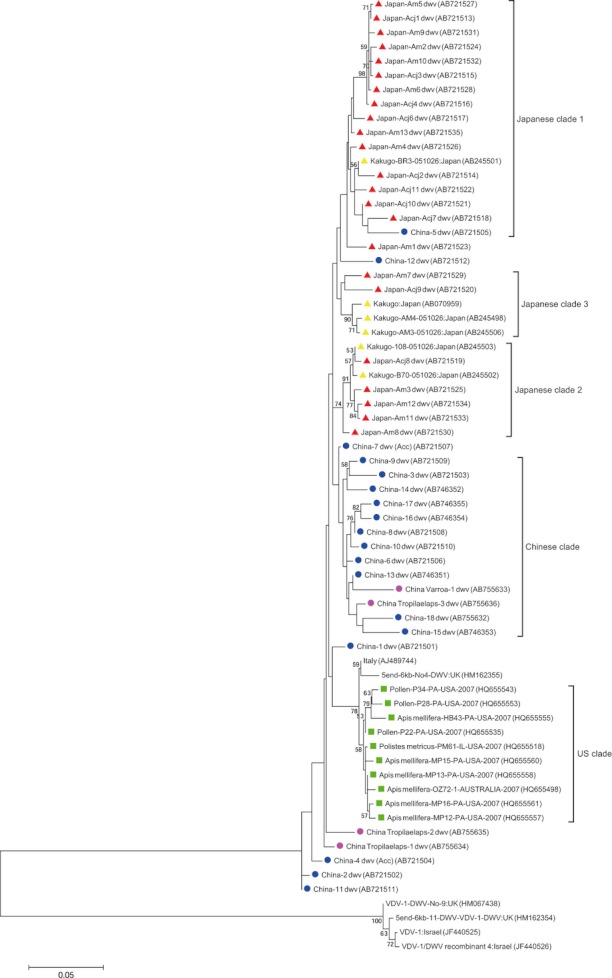
Phylogeny of deformed wing virus (DWV) isolates from China and other countries. The phylogenetic tree based on alignment of the capsid protein gene sequences of DWV isolates from China, Japan, US, UK, and Italy as well as *V. destructor* virus-1 (VDV-1) isolates was constructed using the maximum likelihood method under the Tamura 3-parameter with a discrete gamma distribution and invariable sites model (T92 + G+I). The indicated branching topology was evaluated by bootstrap resampling of the sequences 1000 times, and nodes supported by bootstrap values >50 are shown. Each isolate is indicated by the country of isolation and GenBank accession number. Chinese isolates identified by this study are shown by blue (the isolates from honey bees) and purple (the isolates from mites) circles, and the isolates from *Apis cerana cerana* are indicated by (Acc). Japanese isolates characterized by this and other studies are shown by red and yellow triangles, respectively. The isolates from *A. mellifera ligustica* and *A. c. japonica* are indicated by Am and Acj in the identification labels. The US isolates are indicated by green squares. The Chinese, US, and Japanese clades 1, 2, and 3 are shown by brackets.

**Figure 3:**
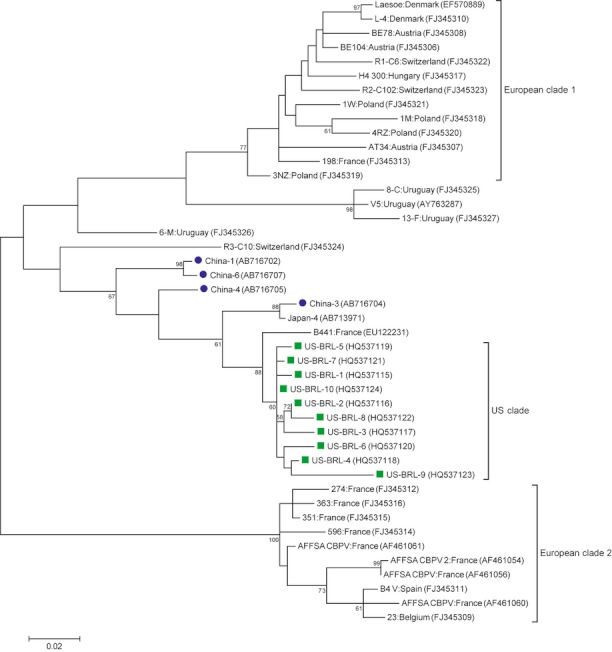
Phylogeny of chronic bee paralysis virus (CBPV) isolates from China and other countries. The phylogenetic tree based on alignment of the putative RdRP gene sequences of CBPV isolates from various countries was constructed using the maximum likelihood method under the Tamura-Nei with a discrete gamma distribution model (TN93 + G). The indicated branching topology was evaluated by bootstrap resampling of the sequences 1000 times, and nodes supported by bootstrap values >50 are shown. Each isolate is indicated by the country of isolation and GenBank accession number. Chinese isolates identified by this study are shown by blue circles, and US isolates are indicated by green squares. US and European clades 1 and 2 are shown by brackets.

**Figure 4:**
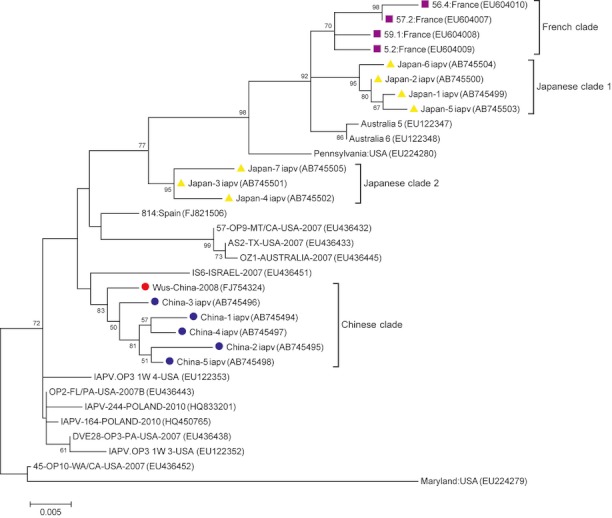
Phylogeny of Israeli acute paralysis virus (IAPV) isolates from China and other countries. The phylogenetic tree based on alignment of a part of RdRP and capsid protein gene sequences of IAPV isolates from various countries was constructed using the maximum likelihood method under the Tamura 3-parameter with a discrete gamma distribution model (T92 + G). The indicated branching topology was evaluated by bootstrap resampling of the sequences 1000 times and nodes supported by bootstrap values >50 are shown. Each isolate is indicated by the country of isolation and GenBank accession number. Chinese isolates identified by this and other studies are shown by blue and red circles, respectively. Japanese and French isolates are indicated by yellow triangles and purple squares, respectively. Chinese, French, and Japanese clades 1 and 2 are shown by brackets.

**Figure 5:**
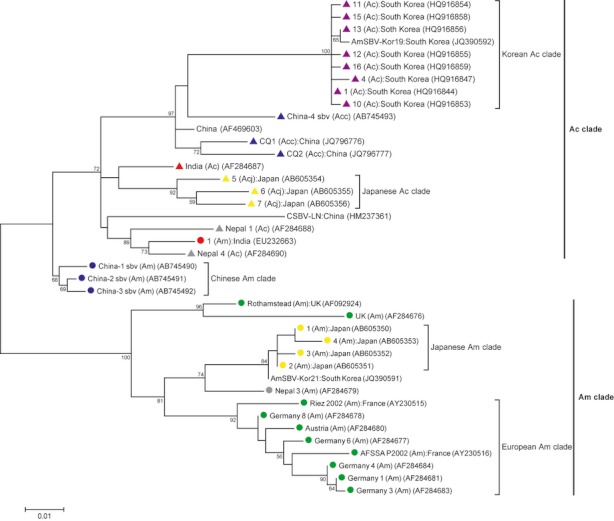
Phylogeny of sacbrood virus (SBV) isolates from China and other countries. The phylogenetic tree based on alignment of the RdRP gene sequences of SBV isolates from various countries was constructed using the maximum likelihood method under the Tamura 3-parameter with invariable sites model (T92 + I). The indicated branching topology was evaluated by bootstrap resampling of the sequences 1000 times and nodes supported by bootstrap values >50 are shown. SBV isolates from *Apis mellifera* and *A. cerana* are indicated by circles and triangles, respectively. The Chinese, Japanese, South Korean, Indian, Nepalese, and European isolates are shown by blue, yellow, purple, red, gray, and green, respectively. When host species are not known, the isolates are not highlighted. Each isolate is indicated by the country of isolation and GenBank accession number. The host species, *A. mellifera*, *A. cerana*, *A. c. cerana*, and *A. c. japonica*, are shown by Am, Ac, Acc, and Acj, respectively, in parentheses. Am and Ac clades are indicated by thick vertical lines. Chinese Am, Japanese Am, European Am, Korean Ac, and Japanese Ac clades are shown by brackets.

**Figure 6:**
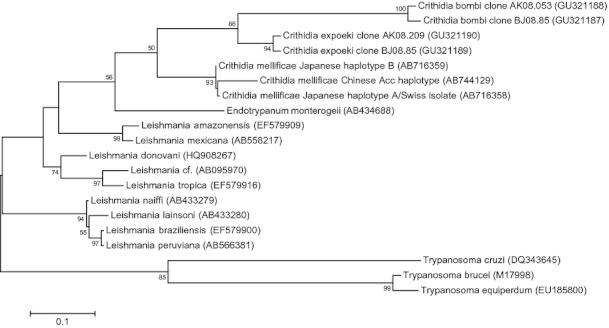
Phylogeny of cytochrome (*Cyt) b* sequences of Chinese and Japanese *Crithidia mellificae* isolates and other trypanosomatids. The phylogenetic tree based on alignment of *Cyt b* sequences of Japanese *C. mellificae* isolates (haplotypes A and B), Chinese isolate from *Apis cerana cerana* (Chinese Acc haplotype), and other trypanosomatids were constructed using the maximum likelihood method under the Tamura-Nei with a discrete gamma distribution model (TN93 + G). The indicated branching topology was evaluated by bootstrap resampling of the sequences 1000 times, and nodes supported by bootstrap values >50 are shown. Each sequence is indicated by organism name and GenBank accession number.

### PCR detection of *N. ceranae* and *N. apis*

Total genomic DNA was isolated from 20 workers collected from single colonies using DNAzol reagent (Invitrogen), and dissolved in 100 μL of 8 mM NaOH followed by neutralization by adding 1 μL of 1 M HEPES. Total DNA (0.1 μg) was used for PCR with KOD FX DNA polymerase and the following primer sets: 5′-CCATTGCCGGATAAGAGAGT-3′ and 5′-CCACCAAAAACTCCCAAGAG-3′ for *N. apis*, and 5′-CGGATAAAAGAGTCCGTTACC-3′ and 5′-TGAGCAGGGTTCTAGGGAT-3′ for *N. ceranae* (Chen et al. 2009). As a control, a honey bee genomic DNA fragment encoding a part of AmHsTRPA (Kohno et al. 2010) was PCR-amplified with the following primers: 5′-CACGACATTCAAGGTTTAAGAAATCACG-3′ and 5′-TCAGTTATTCTTTTCCTTTGCCAGATTT-3′. The thermal cycling conditions and the gel electrophoresis were the same as above. A negative control lacking template DNA and a positive DNA control were performed for each PCR reaction. Positive identification was confirmed by sequencing the PCR products.

### PCR detection of tracheal mite (*A. woodi*)

Total genomic DNA prepared above was used for PCR detection of tracheal mite with the primer set (5′-TCTTCAATTTTAATTATACGT-3′ and 5′-CAAAAATCAGAATAAATGTTGAAATA-3′) as described in (Kojima et al. 2011b). The thermal cycling conditions and the gel electrophoresis were the same as above. A negative control lacking template DNA and a positive DNA control were performed for each PCR reaction. Positive identification was confirmed by sequencing the PCR products.

### PCR detection of *C. mellificae* and *A. bombi*

Total genomic DNA prepared above was used for PCR detection of *C. mellificae* and *A. bombi* as described in (Meeus et al. 2010) with the following primer set: 5′- CTTTTGGTCGGTGGAGTGAT-3′ and 5′-GGACGTAATCGGCACAGTTT-3′ for *C. mellificae*, and 5′-CCAGCATGGAATAACATGTAAGG-3′ and 5′-GACAGCTTCCAATCTCTAGTCG-3′ for *A. bombi*. The thermal cycling conditions and the gel electrophoresis were the same as above. A negative control lacking template DNA and a positive DNA control were performed for each PCR reaction. Positive identification was confirmed by sequencing the PCR products.

### Sequencing of *cytochrome b* gene (*Cyt b*), *small subunit ribosomal RNA* gene (*SSU-rRNA*)*,* and *glyceraldehyde-3-phosphate dehydrogenase (GAPDH) mRNA*

To determine 413-bp DNA sequences of kinetoplast-encoded *Cyt b* of five Chinese *C. mellificae* isolates, genomic PCR products obtained with the primer set 5′-GT(A/T)TT(G/A)TTTTT(G/A)TG(G/A)GATTTTG-3′, and 5′-CATAAACG(T/C)TCACAATAAAATGC-3′ were sequenced. To sequence a 546-bp DNA fragment of *SSU-rRNA* of *C. mellificae* isolate from *A. c. cerana*, the genomic PCR product obtained with the primer set 5′-TGCCATGGCGTTGACGGGAG-3′ and 5′-CCAACAAAAGCCGAAACGGTAGCCT-3′ was sequenced. To determine a 561-bp *GAPDH* mRNA sequence of *C. mellificae* isolate from *A. c. cerana*, an RT-PCR product obtained with the primer set 5′-GGTCGCCGTGGTGGACATGAG-3′ and 5′-AGTCCTTGAGCGACACGCCG-3′ was sequenced.

### Molecular phylogenetic analysis of *Cyt b* sequences

To construct a phylogenetic tree of *Cyt b* sequences from Chinese and Japanese *C. mellificae* isolates and other related trypanosomatids, appropriate *Cyt b* sequences were retrieved from GenBank; these are indicated by organism names with accession numbers. The phylogenetic tree was constructed as above for honey bee viral phylogenies.

## Results and Discussion

### Viruses detected with honey bee colonies in China

The results of virus survey are summarized in Table 2. Viruses were detected in all samples except in the *A. c. cerana* colony from the Guangdong province (GD-2). Multiple viruses were present except in 2 *A. m. ligustica* colonies (YC and GD-1; positive for DWV only) in the virus-positive samples. Such multiple viral infections are quite common with honey bee colonies (e.g., those in Japan and China) (Kojima et al. 2011a; Ai et al. 2012; Morimoto et al. 2012a,b). CBPV was specifically found in dying *A. m. ligustica* workers (worker bees crawling on the ground) at 5 apiaries, SZ-2, NJ-1, NJ-3, NJ-4, and NJ-5, suggesting that CBPV may be responsible for the symptom. This was similar to our previous observation with dying workers infected with CBPV in Japan. However, it remains to be determined whether CBPV actively proliferates in the dying workers by testing the presence of the negative strand of viral RNA genome (Morimoto et al. 2012a). Among the tested samples, DWV was the most prevalent (90%), and neither ABPV nor KBV were detected. In summary, the prevalence and the type of viruses present in *A. m. ligustica* colonies in China appeared to be similar to those in Japan (Kojima et al. 2011a; Morimoto et al. 2012a,b); however, the number of tested colonies in this study is small, demonstrating that the above parameters would change if more colonies were tested. Nevertheless, our results are quite similar to the previous report of virus survey in China (Ai et al. 2012).

**Table 2:** Summary of pathogens and parasites present in honey bee colonies in China

Apiary	Province	Virus	*Nosema* microsporidia	*Crithidia mellificae*	*Apicystis bombi*	*Acarapis woodi*
SZ-1	Jiangsu	BQCV, DWV, IAPV	*N. ceranae*	−	−	−
SZ-2	Jiangsu	BQCV, CBPV (DW), DWV, SBV	*N. ceranae*	−	−	−
SZ-3	Jiangsu	BQCV, DWV	*N. ceranae*	−	−	−
SZ-3 (*A. c. cerana*)	Jiangsu	BQCV, CBPV, DWV	*N. ceranae*	−	−	−
SZ-4	Jiangsu	BQCV, DWV	*N. ceranae*	−	−	−
SZ-5	Jiangsu	BQCV, DWV	–	−	−	−
SZ-5 (*A. c. cerana*)	Jiangsu	BQCV, DWV, SBV	*N. ceranae*	−	−	−
NJ-1	Jiangsu	BQCV, CBPV (DW), DWV	*N. ceranae*	−	−	−
NJ-2	Jiangsu	BQCV, DWV	–	−	−	−
NJ-3	Jiangsu	BQCV, CBPV (DW), DWV, IAPV	–	−	−	−
NJ-4	Jiangsu	BQCV, CBPV (DW), DWV	–	−	−	−
NJ-5	Jiangsu	BQCV, CBPV (DW), DWV	*N. ceranae*	−	−	−
YC	Jiangsu	DWV	–	+	−	+
SH	Shandon	DWV, IAPV	*N. ceranae*	−	−	−
HB	Hubei	BQCV, DWV, IAPV, SBV	*N. ceranae*	+	−	−
SC	Sichuan	BQCV, IAPV	*N. ceranae*	−	−	−
FJ	Fujian	BQCV, DWV, SBV	–	+	−	−
GD-1	Guangdong	DWV	*N. ceranae*	−	−	−
GD-2 (*A. c. cerana*)	Guangdong	–	*N. ceranae*	+	−	−
JL	Jilin	BQCV, DWV	*N. ceranae*	+	−	−

Three *A. c. cerana* colonies are shown by parentheses following the apiary names. The rest of 17 samples are *A. m. ligustica* colonies. DW in parenthesis indicates that pathogens and parasites are specifically detected with dying worker bees (workers crawling on the ground).+ and - indicate positive and negative detections, respectively.

### Molecular phylogenetic characterization of honey bee viruses in China

To infer the phylogeny of Chinese BQCV isolates, we determined the sequences of the capsid protein-coding region of 16 Chinese isolates (AB723728–AB723739 and AB746347–AB746350), and found that eight isolates had sequence variations. These sequences were phylogenetically analyzed along with the 10 newly determined sequences of Japanese BQCV isolates (AB723740–AB723749) and those of the other countries' isolates, as shown in Fig. 1. Although the bootstrap values of many nodes were relatively low (<50), Asian, European, and US isolates tended to separate with a few exceptions. This demonstrated the geographic separation that occurred in BQCV at the continent level. With the exception of one isolate, Japan-Am3_bqcv (AB723742), Japanese isolates were grouped into a single clade (Japanese clade), suggesting that BQCV populations in Japanese apiaries would be relatively homogeneous, having a single origin. In contrast, 10 Chinese isolates from *A. m. ligustica* and *A. c. cerana* clustered into Chinese clade 1 and clade 2; however, the others were dispersed in the phylogeny, suggesting that heterogeneous BQCV populations having multiple origins are present in Chinese apiaries. The US isolates formed two clusters (US clade 1 and clade 2); however, six isolates were not included and dispersed in the phylogeny; therefore, BQCV populations in the US apiaries also appeared to be heterogeneous.

The phylogeny of Chinese DWV isolates was inferred by the 820-nt sequences encoding capsid protein (VP1) of isolates from Japan, US, UK, and Italy, as well as the *V. destructor* virus-1 (VDV-1), as shown in Fig. 2. Similar to BQCV above, the bootstrap values of many nodes are relatively low (<50). Consistent with our previous result obtained with the 393 nt sequences encoding capsid proteins VP2, VP4, and VP1 (Kojima et al. 2011a), Japanese DWV isolates were separated into three major clades (Japanese clade 1, clade 2, and clade 3) in which the isolates from both *A. m. ligustica* and *Apis cerana japonica* were present. All US isolates formed a single cluster (US clade), and, except in one case (China-5_dwv, AB721505 in Japanese clade 1), the US, Japanese, and Chinese isolates did not group together. This demonstrated that DWV had been geographically separated among the above-mentioned countries. Monophyletic clustering of US isolates along with UK and Italian isolates suggests that they share a common origin, and that relatively homogenous populations of DWV are present in US and European apiaries. The introduction of *Varroa* mites in the US in 1987 and its subsequent establishment might result in the spread of a particular strain of DWV, as reported in Hawaii (Martin et al. 2012). Although 13 Chinese isolates were grouped into one clade (Chinese clade), the other nine isolates were dispersed in the phylogeny; therefore, DWV populations in Chinese apiaries still retained some degree of genetic diversity, even though the infestation of both *Varroa* and *Tropilaelaps* mites as well as DWV infection was quite prevalent. The same appears to be the case for Japanese apiaries where *Varroa* mites and DWV are also prevalent (Fig. 2; Kojima et al. 2011a). DWV in Asian countries may keep the genetic diversity because it is able to infect back and forth between nonnative *A. meliifera* and native honey bees, such as *A. cerana*, through their direct interactions or mites infestation.

To analyze the phylogeny of Chinese CBPV isolates, we sequenced a part of the putative RdRP coding sequences of six isolates (AB716702–AB716707) and found that four had sequence variations. The phylogeny of four Chinese isolates and Japanese, European, Uruguayan, and US CBPV isolates is shown in Fig. 3. Except for one Swiss (FJ345324) and one French (EU122231) isolate, the European isolates were grouped into two major clusters (European clades 1 and 2), and the mainland US isolates were clustered as a single clade (US clade), suggesting that each clade shared a common origin. Except for one isolate (FJ345326), Uruguayan isolates also formed an independent clade. European, US, Uruguayan, and Asian (Chinese and Japanese) isolates did not form mixed clades; therefore, they were geographically separated at the continent level; however, the geographic separation was not observed at the country level in European and Asian clades. Four Chinese isolates did not cluster, and one isolate (China-3, AB716704) grouped with the Japanese isolate (Japan-4, AB713971), suggesting that the genetically diverged strains of CBPV from a common origin were spread within Chinese apiaries.

To infer the phylogeny of Chinese IAPV isolates, we determined the 706-nt sequences encoding a part of an RdRP and capsid protein (AB745494–AB745498), and then phylogenetically analyzed them along with the seven newly determined sequences of Japanese isolates (AB745499–AB745505) and those of other countries' isolates. As shown in Fig. 4, our Chinese isolates clustered with another Chinese isolate (FJ754324) to form a monophyletic cluster (Chinese clade), demonstrating that they shared a common origin. Similarly, French isolates also formed an independent clade (French clade). Japanese isolates were grouped into two clusters (Japanese clade 1 and clade 2); therefore, at least two diverged populations of IAPV from a common origin were present in Japanese apiaries. Chinese, French, and Japanese isolates were also geographically separated. Meanwhile, US isolates were dispersed throughout the phylogeny, demonstrating that quite heterogeneous populations of IAPV with diverse origins are present in the US apiaries, as previously suggested (Palacios et al. 2008).

To analyze the phylogeny of four Chinese SBV isolates, we determined the 558-nt sequences encoding RdRP, and then the phylogeny was constructed with those of other countries' isolates from both *A. meliifera* and *A. cerana* (Fig. 5). As we previously showed (Kojima et al. 2011a), SBV sequences were clearly separated into two groups with the isolates from either *A. meliifera* or *A. cerana* (Am clade or Ac clade). One Indian isolate from *A. mellifera* (EU232663) appeared to be the only exception to this rule. This is in large contrast to the phylogeny of DWV in which the isolates from both *A. mellifera* and *A. cerana* were clustered together (Fig. 2); therefore, unlike DWV, SBV might have a species barrier for the infection of *A. mellifera* and *A. cerana*. One of our Chinese SBV isolates from *A. c. cerana* (China-4_sbv, AB745493) and two other Chinese isolates from *A. c. cerana* (JQ796776 and JQ796777) were also included in the Ac clade. Within the Ac clade, Korean and Japanese isolates formed independent clusters (Korean clade and Japanese clade), suggesting that each country's isolates had a common origin and showed geographic separation. Chinese isolates from *A. c. cerana* were not clustered, suggesting that they are genetically heterogeneous, and diverged from the common origin of the Ac clade. In the Am clade, except for two UK isolates (AF092924 and AF284676), Japanese and European isolates were grouped into two clusters (Japanese and European Am clade). Intriguingly, three Chinese SBV isolates from *A. m. ligustica* (AB745490–AB745492) formed a monophyletic group, which did not cluster with the Am clade, and outgroup Ac clade, and thus represented new SBV strains. Relatively homogenous populations of new SBV strain with a single origin appeared to circulate among *A. m. ligustica* colonies in Chinese apiaries.

In summary, genetically heterogeneous populations of BQCV, DWV, CBPV, and *A. cerana*-infecting SBV appear to be present in Chinese apiaries. Multiple strains of BQCV, DWV, and CBPV would have been present when *A. m. ligustica* colonies were introduced into China. Because these viruses also infect *A. c. cerana* (Table 2; Ai et al. 2012), some strains could be originally derived from *A. c. cerana* or possibly other native honey bee species (*Apis florae* and *Apis dorsata*) in China (Zhang et al. 2012). The interactions between *A. m. ligustica* and *A. c. cerana*, through, for example, robber bees, might result in the transmission of such viruses. In contrast to the above viruses, relatively homogenous populations of IAPV and *A. mellifera*-infecting new strain of SBV with single origins appear to be present in Chinese apiaries. This might suggest that the specific fraction of *A. m. ligustica* colonies introduced into China was infected by the above viruses. However, characterization of more isolates may reveal the viral strains with different origins.

### *Nosema* microsporidia detected in honey bee colonies in China

We found that *N. ceranae* was prevalent (70%) in the tested samples including three *A. c. cerana* colonies (SZ-3, SZ-5, and GD-2); however, *N. apis* was not detected in the 20 honey bee colonies we tested (Table 2). This prevalence of *N. ceranae* is similar to the observation with *A. mellifera* colonies in the US, Japan, and several European countries (Fries 2010; Kojima et al. 2011a). Although the role of *N. ceranae* infection in honey bee colony losses remains still unclear (Fries 2010), *N. ceranae* appears to be the dominant *Nosema* microsporidia infecting both native *A. c. cerana* and nonnative *A. m. ligustica* colonies in China. Because both *N. ceranae* and viruses are prevalent, 60% of the tested samples are infected with multiple viruses and *N. ceranae*.

### Protozoan parasite infecting honey bee colonies in China

We found that five colonies (YC, HB, FJ, GD-2, and JL) tested positive for *C. mellificae*, but *A. bombi* was not found among the tested samples (Table 2). Because we did not find any infected *A. c. japonica* colonies in Japan, *C. mellificae* was found for the first time in one *A. c. cerana* colony (GD-2) (Morimoto et al. 2012b). This clearly demonstrates that both *A. mellifera* and *A. cerana* became a host for *C. mellificae* infection.

We then sequenced 413-bp DNA fragments of the kinetoplast-encoded *Cyt b* from five Chinese *C. mellificae* isolates. HB and FJ are identical to Japanese haplotype B, and JL and YC are identical to Japanese haplotype A/Swiss isolate (Schmid-Hempel and Tognazzo 2010; Morimoto et al. 2012b). Japanese haplotypes A and B are different at only two sites (Morimoto et al. 2012b) in the sequence; however, the GD-2 sequence (Chinese Acc haplotype, AB744129) shows the nucleotide differences at 19 sites from both Japanese haplotypes A and B (4.6% sequence divergence). Nevertheless, no differences in the amino acid sequence were found among the three haplotypes; they are clustered and they outgroup other *Crithidia* species based on the phylogeny of *Cyt b* sequences, as shown in Fig. 6. To characterize DNA sequences of nuclear-encoded genes of Chinese Acc haplotype, a 546-bp DNA fragment of *SSU-rRNA* gene was sequenced (AB745487), and one nucleotide change from both Swiss (GU321196, T→C at 448) and Japanese (AB738082, T→C at 429) isolates' sequences was identified. We also determined the 561-bp *GAPDH* mRNA sequence (AB745489), and found that it was the same as the Japanese isolate's sequence (AB716357) with one nucleotide substitution (266C→T) resulting in an amino acid change (89A→V) from a US isolate sequence (JF423199). These results demonstrated that the nuclear-encoded gene sequences of Chinese Acc haplotype are well conserved with those of Swiss, US, and Japanese isolates. The diverged *Cyt b* sequence of the Chinese Acc haplotype suggests that *A. c. cerana* would be the original host. Nevertheless, more honey bee colonies in China need to be tested for *C. mellificae* infection to reveal whether the Chinese Acc haplotype of *C. mellificae* is preferentially detected with *A. c. cerana* relative to *A. m. ligustica*. Furthermore, the degree of virulence of three haplotypes of *C. mellificae* infection in *A. m. ligustica* and *A. c. cerana* remains to be determined.

### *Varroa* and *Tropilaelaps* mites parasitizing honey bee colonies in China

Although we detected only one colony infested by tracheal mites (*A. woodi*) in our honey bee samples (YC, Table 2), both *V. destructor* and *T. mercedesae* were prevalent among *A. m. ligustica* colonies in China (Anderson and Morgan 2007; Luo et al. 2011). This was, in fact, the case for 11 apiaries, SZ-1∼5, NJ-1∼5, and YC, in the Jiangsu province according to questionnaire surveys to the beekeepers. All apiaries had both *Varroa* and *Tropilaelaps* mites in a single hive (*A. m. ligustica*), although the infestation levels appear to change seasonally. The negative impacts of *Varroa* mites on *A. mellifera* colonies have been extensively documented (for example, Rosenkranz et al. 2010); however, the effects of *Tropilaelaps* mites are not well understood because *Tropilaelaps* spp. is still considered to be restricted to Asia (Anderson and Morgan 2007).

**Figure 7:**
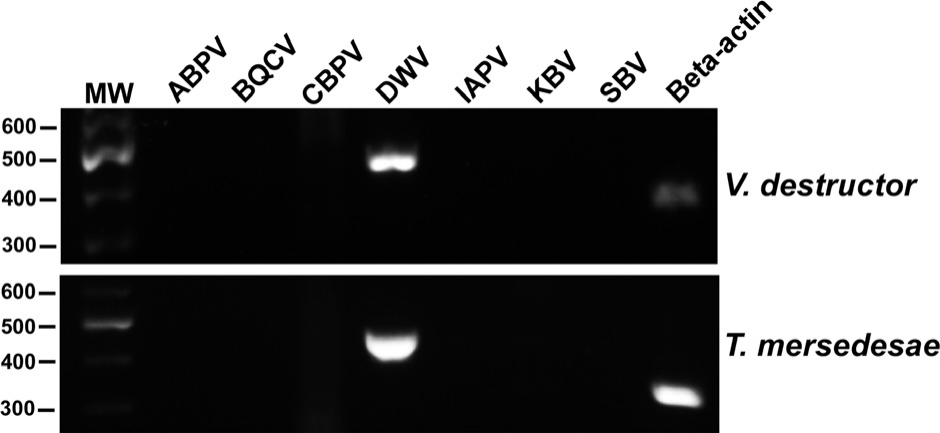
Association of deformed wing virus (DWV) with *Varroa* and *Tropilaelaps* mites in China. Honey bee RNA viruses (acute bee paralysis virus [ABPV], black queen cell virus [BQCV], chronic bee paralysis virus [CBPV], DWV, Israeli acute paralysis virus [IAPV], Kashmir bee virus [KBV], and sacbrood virus [SBV]) associated with *V. destructor* and *T. mersedesae* were tested using RT-PCR of total RNA isolated from the mites. Mite *Beta-actin* mRNA was used as a positive control for RT-PCR. The size of each band with the molecular weight marker (MW) is shown on the left.

To measure the possible impacts of *Tropilaelaps* mites on *A. m. ligustica* colonies in China, we collected *Varroa* and *Tropilaelaps* mites at four apiaries and tested the associated viruses using RT-PCR. Consistent with previous results (Dainat et al. 2009; Forsgren et al. 2009), we found that both mite species harbored DWV in all samples, and the results obtained with *V. destructor* and *T. mercedesae* sampled at apiaries SZ-1 and FJ, respectively, are shown in Fig. 7. Although honey bees (*A. m. ligustica*) from the same hive from which we collected the mites at apiaries SZ-1, SZ-5, and FJ were infected with BQCV, DWV, IAPV, and SBV (Table 2), DWV was the only virus detected in the mites. This might suggest that DWV preferentially associates and proliferates in *Tropilaelaps* mites as in *Varroa* mites (Yue et al. 2007; Gisder et al. 2009); therefore, both *Varroa* and *Tropilaelaps* mites are likely to share the roles for DWV transmission in *A. m. ligustica* colonies in China. Consistent with this idea, the capsid protein gene sequences of two DWV isolates from *V. destructor* and *T. mercedesae,* China Varroa-1 dwv (AB755633) and China Tropilaelaps-3 dwv (AB755636), are clustered within the Chinese clade, but not VDV-1 in phylogeny (Fig. 2), demonstrating that DWV associated with both *Varroa* and *Tropilaelaps* mites were originated from honey bees in China.
